# MONARCH 3 final PFS: a randomized study of abemaciclib as initial therapy for advanced breast cancer

**DOI:** 10.1038/s41523-018-0097-z

**Published:** 2019-01-17

**Authors:** Stephen Johnston, Miguel Martin, Angelo Di Leo, Seock-Ah Im, Ahmad Awada, Tammy Forrester, Martin Frenzel, Molly C. Hardebeck, Joanne Cox, Susana Barriga, Masakazu Toi, Hiroji Iwata, Matthew P. Goetz

**Affiliations:** 1 Breast Unit, The Royal Marsden NHS Foundation Trust, London, UK; 2 Instituto De Investigacion Sanitaria Gregorio Marañon, Ciberonc, Geicam; Universidad Complutense, Madrid, Spain; 3 Department of Oncology, Hospital of Prato, Istituto Toscano Tumori, Prato, Italy; 4 Department of Internal Medicine, Seoul National University Hospital, Cancer Research Institute, Seoul National University College of Medicine, Seoul, Korea; 5 Oncology Medicine department, Jules Bordet Institute, Université Libre de Bruxelles, Brussels, Belgium; 6 Global Statistical Sciences, Eli Lilly and Company, Indianapolis, IN USA; 7 Oncology Clinical Development, Eli Lilly and Company, Indianapolis, IN USA; 8 Oncology Clinical Development, Eli Lilly and Company, Windlesham, UK; 9 Oncology Clinical Development, Eli Lilly and Company, Madrid, Spain; 10 Department of Breast Surgery, Kyoto University, Kyoto, Japan; 11 Department of Breast Oncology, Aichi Cancer Center Hospital, Nagoya, Japan; 12 Department of Oncology, Mayo Clinic, Rochester, MN USA

## Abstract

At the MONARCH 3 interim analysis, abemaciclib plus a nonsteroidal aromatase inhibitor (AI) significantly improved progression-free survival (PFS) and objective response rate (ORR) with a tolerable safety profile as initial treatment for hormone receptor-positive (HR+), human epidermal growth factor receptor 2-negative (HER2−) advanced breast cancer (ABC). MONARCH 3 is a randomized, phase III, double-blind study of abemaciclib/placebo (150 mg twice daily, continuous) plus nonsteroidal AI (1 mg anastrozole or 2.5 mg letrozole, daily). A total of 493 postmenopausal women with HR+, HER2− ABC with no prior systemic therapy in this setting were enrolled. The primary endpoint was investigator-assessed PFS (final analysis after 240 events); other endpoints included response and safety evaluations. Here we analyze the final PFS data and update secondary endpoints. The abemaciclib arm had a significantly longer median PFS than the placebo arm (28.18 versus 14.76 months; hazard ratio [95% confidence interval], 0.540 [0.418–0.698]; *p* = .000002). The ORR was 61.0% in the abemaciclib arm versus 45.5% in the placebo arm (measurable disease, *p* = .003). The median duration of response was longer in the abemaciclib arm (27.39 months) compared to the placebo arm (17.46 months). The safety profile was consistent with previous reports. The most frequent grade ≥ 3 adverse events in the abemaciclib versus placebo arms were neutropenia (23.9% versus 1.2%), diarrhea (9.5% versus 1.2%), and leukopenia (8.6% versus 0.6%). Abemaciclib plus a nonsteroidal AI was an effective initial treatment with an acceptable safety profile for HR+, HER2− ABC.

## Clinical trial: Combo of cell cycle blocker and hormonal therapy proves highly effective

Patients with the most common form of breast cancer stand to benefit from taking a drug that blocks two cell cycle–regulating proteins in addition to hormonal therapy. In a phase III clinical trial, Stephen Johnston from the Royal Marsden NHS Foundation Trust in London, UK, and colleagues randomly gave 493 postmenopausal women with HR+/HER2− metastatic breast cancer a nonsteroidal aromatase inhibitor plus either a placebo or a drug called abemaciclib, a targeted inhibitor of CDK4 and CDK6. A planned interim analysis previously showed that abemaciclib was safe and effective. Johnson’s team now reports that abemaciclib nearly doubled the time women live without disease recurrence, from 15 months on placebo to 28 months on the drug. Additionally, responses were more common and lasted longer among women who received both abemaciclib and the hormonal therapy.

## Introduction

Sequential endocrine therapy regimens comprise the standard of care for hormone receptor-positive (HR+) advanced breast cancer.^1,2^ However, endocrine therapy resistance remains a major clinical challenge, as patients ultimately succumb to progressive disease.^3,4^ New therapeutic strategies that synergize with endocrine therapies are needed to overcome resistance and potentially prolong patient survival. The primary clinical endpoint to measure resistance to endocrine therapies is progression-free survival (PFS), and improvements in PFS can be associated with overall survival in advanced breast cancer.^5,6^ However, tumor shrinkage, specifically objective response rate (ORR), is also associated with overall survival in this disease.^7,8^ Thus, improving the frequency, depth, and duration of response of endocrine-based therapies while overcoming resistance to endocrine therapy may ultimately translate into prolonged survival.

Inhibition of cyclin dependent kinase 4 (CDK4) and cyclin dependent kinase 6 (CDK6) has shown considerable promise in attenuating endocrine therapy resistance.^9,10^ CDK4 and CDK6 are not only essential for the G1 to S phase cell cycle transition, but also play a central role in the growth of HR+ breast cancer cells.^11–14^ Thus, in the clinic, CDK4 and CDK6 inhibition has been an effective means to treat HR+, HER2− advanced breast cancer; currently, three CDK4 and CDK6 inhibitors (palbociclib, ribociclib, and abemaciclib) have been approved in combination with endocrine therapy for the treatment of this disease, based on improvements in PFS and ORR.^15–17^

Abemaciclib is an orally administered CDK4 and CDK6 inhibitor that is 14 times more potent against CDK4/cyclin D1 than CDK6/cyclin D3 (based on enzymatic assays) and, unlike other currently approved CDK4 and CDK6 inhibitors, is dosed on a twice daily continuous schedule.^13,15–17^ In preclinical models, continuous inhibition by abemaciclib was essential for promoting sustained cell cycle arrest, leading to apoptosis or senescence, while short-term inhibition led to a rebound effect upon withdrawal.^13,18^ Furthermore, preclinical evidence has demonstrated a potential novel role for abemaciclib in promoting anti-tumor immunity through increased antigen presentation and selective inhibition of regulatory T-cell proliferation, possibly suggesting that abemaciclib may be operating through multiple mechanisms to induce tumor shrinkage.^19^

Abemaciclib was approved in September 2017 by the U.S. Food and Drug Administration (FDA) in combination with fulvestrant for women with HR+, HER2− advanced or metastatic breast cancer that progressed following endocrine therapy, based on significant improvements in PFS and ORR observed in the MONARCH 2 trial.^9,17^ In addition, abemaciclib is the only CDK4 and CDK6 inhibitor currently approved by the FDA as monotherapy for patients with heavily pretreated HR+, HER2− metastatic breast cancer based on single agent ORR observed in the MONARCH 1 trial.^17,20^ Abemaciclib has now been approved by the FDA (February 2018) in combination with a nonsteroidal aromatase inhibitor (AI) based on the MONARCH 3 trial (NCT02246621). At the preplanned interim analysis of the MONARCH 3 trial, abemaciclib in combination with a nonsteroidal AI demonstrated significant improvements in PFS and ORR with a generally tolerable safety profile as initial therapy for patients with HR+, HER2− advanced breast cancer.^21^

Here we present the preplanned final analysis of PFS for the MONARCH 3 trial along with detailed analyses of endpoints associated with objective response, including tumor shrinkage and duration of response. Overall, these analyses demonstrate that abemaciclib in combination with a nonsteroidal AI prolonged PFS; improved the frequency, depth, and duration of response; and provided a tolerability profile consistent with other breast cancer studies of abemaciclib.

## Results

### Patients and treatment

From November 18, 2014 to November 11, 2015, a total of 493 patients were randomly assigned to abemaciclib plus a nonsteroidal AI (*n* = 328) or placebo plus a nonsteroidal AI (*n* = 165) (Supplementary Fig. S1). Baseline patient and disease characteristics were similar between treatment arms and were previously described.^21^ Briefly, approximatively 80% of patients presented with measurable disease at baseline and 196 (39.8%) patients had de novo metastatic breast cancer. A total of 230 (46.7%) patients had received prior neoadjuvant or adjuvant endocrine therapy, and 191 (38.7%) had received prior neoadjuvant or adjuvant chemotherapy.

At the final PFS cutoff on November 3, 2017, a total of 125 (38.1%) patients in the abemaciclib arm and 35 (21.2%) patients in the placebo arm remained on treatment (Supplementary Fig. S1). The median number of cycles received was 19 in the abemaciclib arm and 15 in the placebo arm. The median relative dose intensity was 85.25% for abemaciclib compared to 98.25% for placebo.

### Efficacy

**Fig. 1:**
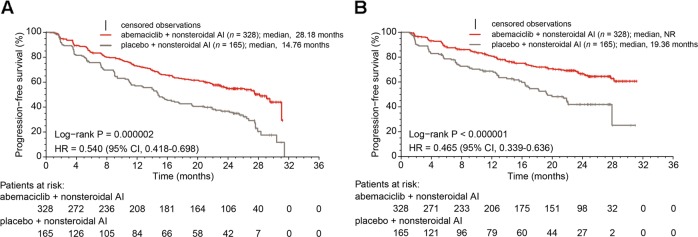
Progression-free survival. **a** Investigator-assessed and **b** Independent central review in the intent-to-treat population. NR, not reached

**Fig. 2:**
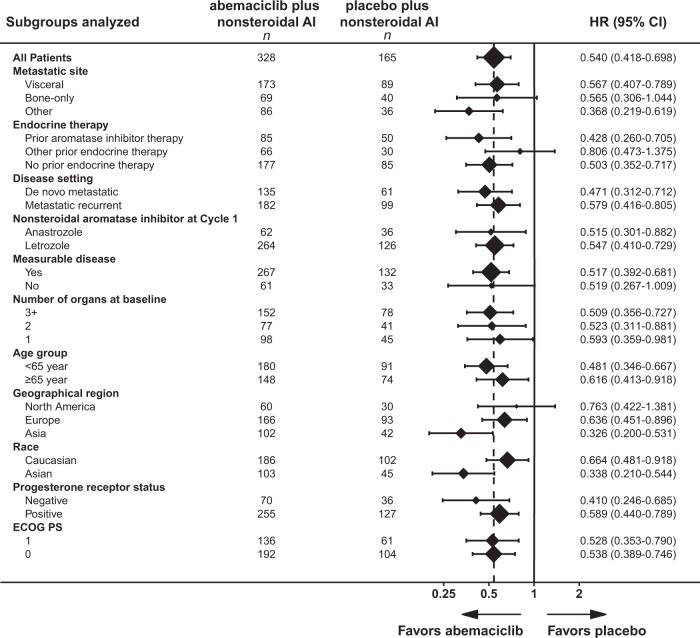
Subgroup analysis of progression-free survival. ECOG PS, Eastern Cooperative Oncology Group Performance Status

At the final data cutoff, 246 PFS events had occurred, of which 138 (42.1%) were in the abemaciclib arm and 108 (65.5%) were in the placebo arm. The median follow-up was 26.73 months. The median investigator-assessed PFS was 28.18 months in the abemaciclib arm versus 14.76 months in the placebo arm (hazard ratio [HR] [95% confidence interval (CI)]: 0.540 [0.418 to 0.698]; *p* = .000002) (Fig. 1a). These PFS results were consistent with the independent central review assessment of PFS (HR [95% CI]: 0.465 [0.339 to 0.636]; *p* < .000001) (Fig. 1b). Analysis of PFS across patient subgroups demonstrated that all patient subgroups benefited from the addition of abemaciclib to nonsteroidal AI (Fig. 2).

**Fig. 3:**
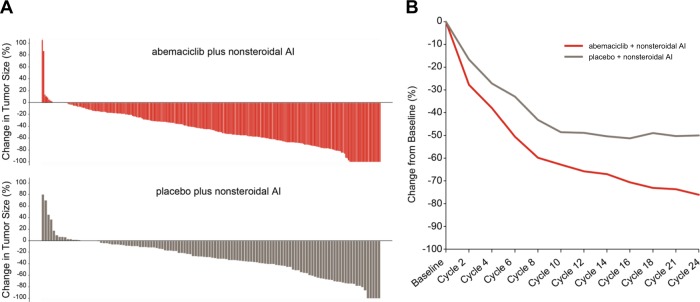
Tumor response for patients with measurable disease. **a** Best percent change in tumor size. **b** Mean: percentage change in tumor size from baseline by cycle

For the intent-to-treat (ITT) population, the ORR was 49.7% (95% CI 44.3–55.1%) in the abemaciclib arm and 37.0% (95% CI 29.6–44.3%) in the placebo arm (*p* = .005) (Table 1). Patients with bone-only non-measurable disease cannot have a best response of partial response; thus, the remaining analyses focus on a subset of 399 patients (80.9%) with measurable disease. In patients with measurable disease, the ORR was 61.0% (95% CI 55.2–66.9%) in the abemaciclib arm versus 45.5% (95% CI 37.0–53.9%) in the placebo arm (*p* = .003), including 9 (3.4%) complete responses in the abemaciclib arm and none in the placebo arm (Table 1). The best percentage change in tumor size for patients with measurable disease was greater for those in the abemaciclib arm than in the placebo arm (Fig. 3a). The median time to initial response was similar between arms (3.58 months in the abemaciclib arm and 3.65 months in the placebo arm). However, an exploratory analysis of mean change in tumor size demonstrated that after 2 cycles, tumor size in the abemaciclib arm decreased by 27.7% compared to 16.6% in the placebo arm. Tumor shrinkage in the abemaciclib arm continued for at least 24 cycles (approximately 22 months), whereas tumor shrinkage in the placebo arm plateaued after approximately 10–12 cycles of treatment (Fig. 3b). Following 24 cycles of treatment, the mean decrease in tumor size was 76.1% in the abemaciclib arm and 50.0% in the placebo arm.

**Table 1:** Best overall response

Best overall response^a^	Abemaciclib plus nonsteroidal AI		Placebo plus nonsteroidal AI		*p* value
	*n* (%)	95% CI	*n* (%)	95% CI	
All patients, *n*	328		165		
CR	9 (2.7)	1.0, 4.5	1 (0.6)	-0.6, 1.8	
PR	154 (47.0)	41.6, 52.4	60 (36.4)	29.0, 43.7	
SD	128 (39.0)	33.7, 44.3	82 (49.7)	42.1, 57.3	
SD ≥ 6 months	93 (28.4)	23.5, 33.2	57 (34.5)	27.3, 41.8	
Progressive disease	12 (3.7)	1.6, 5.7	12 (7.3)	3.3, 11.2	
Not evaluable	25 (7.6)	4.8, 10.5	10 (6.1)	2.4, 9.7	
Objective response rate (CR/PR)^b^	163 (49.7)	44.3, 55.1	61 (37.0)	29.6, 44.3	0.005
Disease control rate (CR/PR/SD)	291 (88.7)	85.3, 92.1	143 (86.7)	81.5, 91.9	0.501
Clinical benefit rate (CR/PR/SD ≥ 6 months)	256 (78.0)	73.6, 82.5	118 (71.5)	64.6, 78.4	0.101
Patients with measurable disease at baseline, *n*	267		132		
CR	9 (3.4)	1.2, 5.5	0	N/A	
PR	154 (57.7)	51.8, 63.6	60 (45.5)	37.0, 53.9	
SD	76 (28.5)	23.1, 33.9	54 (40.9)	32.5, 49.3	
SD ≥ 6 months	48 (18.0)	13.4, 22.6	32 (24.2)	16.9, 31.6	
Progressive disease	10 (3.7)	1.5, 6.0	12 (9.1)	4.2, 14.0	
Not evaluable	18 (6.7)	3.7, 9.7	6 (4.5)	1.0, 8.1	
Objective response rate (CR/PR)^c^	163 (61.0)	55.2, 66.9	60 (45.5)	37.0, 53.9	0.003
Disease control rate (CR/PR/SD)	239 (89.5)	85.8, 93.2	114 (86.4)	80.5, 92.2	0.310
Clinical benefit rate (CR/PR/SD ≥ 6 months)	211 (79.0)	74.1, 83.9	92 (69.7)	61.9, 77.5	0.037

N/A, not applicable

^a^Using RECIST v1.1

^b^Confirmed ORR for all patients: 45.1% in the abemaciclib arm, 32.7% in the placebo arm

^c^Confirmed ORR for patients with measurable disease: 55.4% in the abemaciclib arm, 40.2% in the placebo arm

**Fig. 4:**
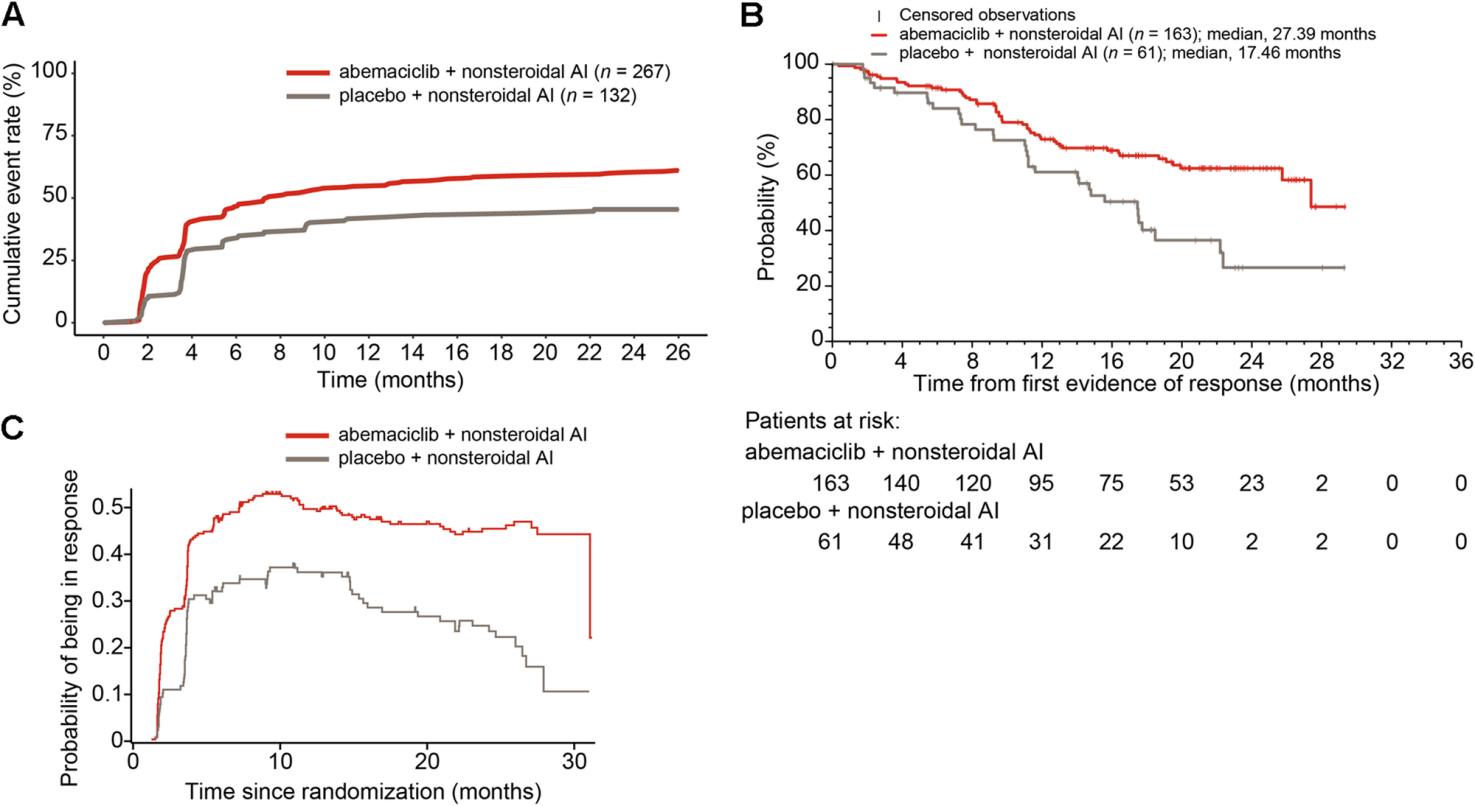
Patient response to treatment. **a** Median time to response for patients with measurable disease (investigator assessment). **b** Duration of response. **c** Probability of response in patients with measurable disease

For patients who responded to abemaciclib, the response typically occurred within the first 8 months of treatment (Fig. 4a). Median duration of response was considerably longer in the abemaciclib arm (27.39 months) than in the placebo arm (17.46 months) (Fig. 4b). Duration of response curves cannot be directly compared by traditional statistical methods, and thus were compared by the probability of being in response function (PBRF) and expected duration of response (EDoR) methods.^22^ The PBRF provides an estimate of the proportion of patients in response over time, presenting simultaneously the time to response and the duration of response for patients with measurable disease. The rate at which the curve initially rises corresponds to the time it takes to obtain a response, confirming a similar time to response in both treatment arms (Fig. 4c). The maximum probability of being in response was reached at approximately 9 months after randomization in the abemaciclib arm and approximately 11 months after randomization in the placebo arm; at these times, the maximum probability of being in response was 53.4% in the abemaciclib arm and 38.1% in the placebo arm. Finally, the rate of decrease of the curve was slower in the abemaciclib arm compared to the placebo arm, suggesting more durable responses in the abemaciclib arm. Approximately two years after randomization, out of 112 patients with measurable disease, almost twice as many patients in the abemaciclib arm were in a responding state compared to placebo, 45.5% versus 23.5%, respectively, a difference considerably larger than the observed difference in the ORR. The EDoR were estimated to 21.4 months in the abemaciclib arm and 9.0 months in the placebo arm. The EDoR ratio for abemaciclib compared to placebo was 2.39 (*p* < 0.001), indicating that the EDoR was significantly higher for patients receiving abemaciclib.

### Safety profile

The safety profile was consistent with the interim analysis (Table 2). In the abemaciclib and placebo arms, 15 deaths (4.6%) (11 due to adverse events) and 3 (1.9%) (2 due to adverse events), respectively, occurred either while patients were receiving therapy or within 30 days of treatment discontinuation. Of these, 3 occurred in the abemaciclib arm after the interim analysis cutoff (lung infection [*n* = 1], respiratory failure [*n* = 1], cerebrovascular accident [*n* = 1]) (Table 2).

**Table 2:** Treatment-emergent adverse events

≥15% occurrence in abemaciclib arm, *n* (%)	Abemaciclib plus nonsteroidal AI (*n* = 327)				Placebo plus nonsteroidal AI (*n* = 161)			
	All grades	Grade 2	Grade 3	Grade 4	All Grades	Grade 2	Grade 3	Grade 4
Any adverse event	323 (98.8)	102 (31.2)	169 (51.7)	22 (6.7)	152 (94.4)	70 (43.5)	36 (22.4)	4 (2.5)
Diarrhea	269 (82.3)	99 (30.3)	31 (9.5)	0	52 (32.3)	14 (8.7)	2 (1.2)	0
Neutropenia	143 (43.7)	53 (16.2)	72 (22.0)	6 (1.8)	3 (1.9)	1 (0.6)	1 (0.6)	1 (0.6)
Fatigue	135 (41.3)	59 (18.0)	6 (1.8)	–	54 (33.5)	21 (13.0)	0	–
Nausea	135 (41.3)	40 (12.2)	4 (1.2)	–	33 (20.5)	1 (0.6)	2 (1.2)	–
Anemia	103 (31.5)	49 (15.0)	23 (7.0)	0	13 (8.1)	3 (1.9)	2 (1.2)	0
Abdominal pain	102 (31.2)	24 (7.3)	6 (1.8)	–	21 (13.0)	6 (3.7)	2 (1.2)	–
Vomiting	99 (30.3)	28 (8.6)	5 (1.5)	0	21 (13.0)	2 (1.2)	4 (2.5)	0
Alopecia	90 (27.5)	7 (2.1)	–	–	18 (11.2)	0	–	–
Decreased appetite	86 (26.3)	30 (9.2)	5 (1.5)	0	17 (10.6)	3 (1.9)	1 (0.6)	0
Leukopenia	72 (22.0)	31 (9.5)	27 (8.3)	1 (0.3)	4 (2.5)	1 (0.6)	0	1 (0.6)
Blood creatinine increased	67 (20.5)	25 (7.6)	6 (1.8)	1 (0.3)	7 (4.3)	1 (0.6)	0	0
Headache	65 (19.9)	11 (3.4)	3 (0.9)	–	26 (16.1)	6 (3.7)	0	–
ALT increased	57 (17.4)	16 (4.9)	20 (6.1)	1 (0.3)	12 (7.5)	3 (1.9)	3 (1.9)	0
Arthralgia	57 (17.4)	14 (4.3)	0	–	33 (20.5)	7 (4.3)	0	–
Constipation	57 (17.4)	12 (3.7)	2 (0.6)	0	23 (14.3)	5 (3.1)	0	0
AST increased	55 (16.8)	15 (4.6)	12 (3.7)	0	12 (7.5)	2 (1.2)	2 (1.2)	0
Back pain	52 (15.9)	18 (5.5)	3 (0.9)	–	26 (16.1)	10 (6.2)	1 (0.6)	–
Rash	50 (15.3)	11 (3.4)	3 (0.9)	0	8 (5.0)	2 (1.2)	0	0

Deaths due to AEs: abemaciclib arm: lung infection (*n* = 4), embolism (*n* = 2), respiratory failure (*n* = 2), cerebral ischemia (*n* = 1), cerebrovascular accident (*n* = 1), pneumonitis (*n* = 1); placebo arm: general physical health deterioration (*n* = 1), sudden death (*n* = 1)

*ALT* alanine aminotransferase, *AST* aspartate aminotransferase

In the abemaciclib arm, longer follow-up confirmed that diarrhea was typically of low grade (72.8% grade 1 or 2) and often occurred early (69.1% of patients experienced diarrhea in cycle 1). The management of diarrhea appeared generally successful with the incidence dropping below 10% (grade 2) and 1% (grade 3) by cycle 4 (Supplementary Fig. S2). Of the patients who had diarrhea, 74.3% in abemaciclib arm received antidiarrheal medications compared to 30.8% in placebo arm. Diarrhea was managed with dose adjustments: 16.7% of patients in abemaciclib arm had a dose reduction and 19% had a dose omission due to diarrhea. Treatment duration was typically not limited by diarrhea, with 6 (1.8%) of the patients in the abemaciclib arm discontinuing treatment for this event (Supplementary table S1). Of note, 4 of these patients were not given a dose reduction prior to discontinuation. The final analysis confirmed that 43.7% of patients had an adverse event of neutropenia, and grade 3 or 4 neutropenia occurred in 23.9% of patients. While one patient experienced an adverse event of febrile neutropenia at the interim analysis, no new cases were observed at this final analysis.

In this study, there was an imbalance of venous thromboembolic events (VTEs) (abemaciclib arm, *n* = 20 [6.1%]; placebo arm, *n* = 1 [0.6%]). Of these, 4 were reported after the interim analysis (all non-serious grade 2 deep vein thromboses).

As would be expected with longer follow-up and the continuous dosing schedule of abemaciclib, dose modification rates were slightly higher compared to the interim analysis. In the abemaciclib versus placebo arms, 152 (46.5%) patients versus 10 (6.2%) patients, respectively, had at least one dose reduction of abemaciclib/placebo due to an adverse event. A total of 82 (25.1%) patients in abemaciclib arm and 7 (4.3%) patients in placebo arm discontinued any study drug as the result of AE. Discontinuation was not due to any specific single adverse event or group of adverse events (Supplementary table S1). Discontinuation of all study treatment due to an adverse event occurred in 54 (16.5%) patients in the abemaciclib arm and 5 (3.1%) in the placebo arm (Supplementary table S1). Furthermore, discontinuation of abemaciclib/placebo (with continuation of nonsteroidal AI) due to an adverse event occurred in 31 (9.5%) patients in the abemaciclib arm and none in the placebo arm. Notably, half of the patients in the abemaciclib arm who discontinued due to an adverse event did not first have a dose reduction (Supplementary Table S2). Conversely, in the abemaciclib arm, 112 (74%) of 152 patients who had a dose reduction due to an adverse event did not discontinue due to toxicity.

### Relationship between early diarrhea and PFS

An exploratory analysis was performed to examine the potential relationship between early toxicities associated with abemaciclib and PFS of patients. Compared to the placebo arm, patients treated with abemaciclib who had diarrhea within the first 7 days (HR [95% CI]: 0.49 [0.35–0.67]) or who did not have diarrhea within the first 7 days (HR [95% CI]: 0.58 [0.43–0.78]) had an improvement in PFS (Supplementary Fig. S3). A time-dependent covariate analysis was performed to examine the association between current dose level (150, 100, and 50 mg) and PFS. Compared to being treated at the 150 mg dose level, there was no apparent difference in PFS for patients reduced to 100 mg (HR [95% CI]: 0.764 [0.467–1.251]; *p* = 0.2849) or to 50 mg (HR [95% CI]: 0.985 [0.511–1.902]; *p* = 0.9650) (Supplementary Table S3).

## Discussion

Analysis from the preplanned final data cutoff of the MONARCH 3 trial provided efficacy and safety results consistent with the interim analysis. The final PFS data confirmed that abemaciclib dosed on a continuous schedule in combination with a nonsteroidal AI provided statistically significant increases in median PFS and ORR as an initial treatment for postmenopausal women with HR+, HER2− advanced breast cancer. The addition of abemaciclib to a nonsteroidal AI provided PFS benefit across all pre-specified subgroups. Importantly, the responses observed in patients who received abemaciclib generally occurred early, were maintained over time, and led to substantial tumor shrinkage. This activity could be relevant for patients with a large tumor burden.

The addition of abemaciclib to a nonsteroidal AI significantly increased the frequency, depth, and duration of tumor response. However, it is difficult to statistically interpret comparisons of duration of response, which considers the length of response only for the responders in each arm, and thus leads to a potential imbalance of clinical characteristics.^22^ To address the bias of this approach, the PBRF method can be used to assess the probability of a patient in each arm being in a responding state at any given time after randomization. This methodology was previously shown to be a useful way of comparing response duration of endocrine therapy for advanced breast cancer.^23^ Here, we found that patients in the abemaciclib arm had an expected duration of response of 21.4 months compared to 9.0 months in the placebo arm. The duration of response in patients treated with abemaciclib was substantiated by the greater than 75% reduction in tumor size observed after 24 cycles.

The safety profile observed at the final PFS cutoff was largely consistent with the interim analysis and with other abemaciclib studies.^9,20,21^ Although discontinuations due to adverse events were higher in the abemaciclib arm compared to the placebo arm, there was not any single adverse event or group of adverse events responsible for the increased number of discontinuations during the preplanned final PFS analysis. Furthermore, half of the discontinuations occurred without first having a dose reduction. Of note, the majority of patients who had a dose reduction due to an adverse event did not discontinue therapy. Importantly, in the exploratory analysis, there was no apparent loss of efficacy in patients who had a dose reduction due to an adverse event, thus highlighting the importance of finding the optimal dose for each patient experiencing an adverse event, such that toxicities can be managed while seeking to preserve efficacy.

Recently, a systematic review and meta-analysis of randomized controlled trials demonstrated that the combination of CDK4 and 6 inhibitors to endocrine therapy (letrozole or fulvestrant) was associated with a higher incidence of VTE.^24^ An increase of VTE was observed in the abemaciclib arm in the registration trials MONARCH 2^9^ and MONARCH 3.^21^ In the abemaciclib arm of MONARCH 3, most of the patients who experienced a VTE were treated with standard anticoagulant medication (17 out of 20). Of the patients who had a VTE, 15 were able to continue therapy.

As reported in the interim analysis, three patients experienced a VTE with a fatal outcome, in one of which the diagnosis was confirmed by CT scan—this patient who underwent a surgery two months prior to starting study treatment, had a pulmonary embolism (PE) and deep vein thrombosis (DVT). One patient with a prior history of PE, hypertension, diabetes, and multiple metastatic lesions experienced respiratory failure, and the cause of death was reported as PE, respiratory failure, and breast cancer. A third patient who was a current tobacco user with history of hypertension, sinus tachycardia, and coronary heart disease experienced a thromboembolism with no further information provided.

As previously reported, diarrhea was generally low grade, typically occurred early in the course of treatment, and in most cases could be effectively managed with antidiarrheal medication and/or dose adjustment if needed.^21^ No difference in efficacy was observed in patients who experienced diarrhea within the first seven days of treatment compared to those who did not.

Abemaciclib in combination with a nonsteroidal AI had a generally predictable and acceptable tolerability profile. When toxicities are experienced by patients, “one size fits all dosing” often is not an optimal way to administer anticancer medicines. Therefore, it is important to tailor the dose of any medicine to the individual patient by observing early toxicities and making the appropriate dose adjustments at that time. When adopting this approach it is beneficial when the toxicities tend to manifest early and are typically rapidly reversible. The diarrhea induced by abemaciclib is such a toxicity. This analysis suggests that patients who received appropriate early dose reductions of abemaciclib could generally remain on therapy and achieved similar benefit to those who did not require dose reduction.

The results of this trial come in light of recent studies demonstrating a mechanism for CDK4/cyclin D in the regulation of cancer immune surveillance.^25^ In addition, the role of abemaciclib in promoting immunogenicity of tumor cells was recently proposed.^19^ Of note, abemaciclib can induce intra-tumor immune inflammation, and the combination of abemaciclib with anti-PD-L1 immunotherapy increased tumor regression in various murine models.^19,26^ These novel findings suggest that CDK4 and CDK6 inhibitors may be operating via additional mechanisms rather than simply inhibiting cell cycle proliferation and could potentially be playing a critical role in the robust tumor response achieved with abemaciclib in this and other studies. Future studies will be necessary to understand how the implications of these findings may affect treatment strategies.

Overall, this preplanned final PFS analysis of MONARCH 3 confirmed that abemaciclib dosed on a continuous schedule in combination with a nonsteroidal AI significantly improved PFS and ORR compared to placebo plus a nonsteroidal AI as initial therapy for patients with HR+, HER2- advanced breast cancer. Patients treated with abemaciclib plus a nonsteroidal AI experienced a longer duration of response. The safety profile was consistent with other abemaciclib studies, indicating that the combination was generally tolerable and manageable.^1,9,20^ Thus, abemaciclib in combination with a nonsteroidal AI was an effective treatment option for patients in this disease setting.

## Materials and methods

### Study design and patients

MONARCH 3 is a randomized, double-blind, phase III trial of abemaciclib or placebo with a nonsteroidal AI (anastrozole or letrozole, per physician’s choice) in postmenopausal women with HR+, HER2− locoregionally recurrent breast cancer not amenable to surgical resection or radiotherapy with curative intent or metastatic disease who have not received prior systemic therapy in the advanced setting. Endocrine therapy in the neoadjuvant or adjuvant setting was permitted if the disease-free interval was greater than 12 months from the completion of endocrine therapy. Detailed inclusion and exclusion criteria were previously described.^21^ Eligible patients were enrolled at 158 sites in 22 countries.

The MONARCH 3 study was approved by the ethical and local institutional review boards for the sites participating in the clinical trial, and was conducted according to the Declaration of Helsinki. Patients provided written informed consent before enrollment. This study was overseen by a steering committee, and safety data were evaluated quarterly by an independent data monitoring committee.

### Treatment

Eligible patients were randomly assigned to receive abemaciclib on a continuous schedule (150 mg, twice daily) plus nonsteroidal AI (1 mg anastrozole or 2.5 mg letrozole, physician’s choice, daily) or placebo plus nonsteroidal AI in a 2:1 ratio using an interactive, web-based randomization scheme. All drugs were orally administrated during each 28-day cycle. Patients were randomly assigned based on stratification factors: metastatic site (visceral, bone-only, or other) and prior neoadjuvant or adjuvant endocrine therapy (AI, no endocrine therapy, or other). Other treatment procedures were previously described.^21^

### Efficacy and safety assessments

Tumors were assessed by computed tomography or magnetic resonance imaging according to RECIST version 1.1 at baseline (within 28 days before randomization) and every second cycle during cycles 2–18, every third cycle thereafter, and within 2 weeks of clinical progression.

Safety analysis was performed on the safety population defined as all enrolled patients receiving at least one dose of study treatment. Adverse events were graded according to National Cancer Institute Common Terminology Criteria version 4.0 and were evaluated at every patient visit from baseline to follow-up. Laboratory analyses schedules were previously described.^21^

### Endpoints

The primary endpoint was investigator-assessed PFS as defined by RECIST version 1.1, and measured from the time of randomized assignment until progressive disease or death from any cause. Secondary endpoints included ORR (percentage of patients with complete response [CR] or partial response [PR]), disease control rate (percentage of patients with CR, PR, or stable disease [SD]), clinical benefit rate (the percentage of patients with CR, PR, or SD ≥ 6 months), duration of response (time from a confirmed CR or PR until disease progression or death), overall survival (time of randomized assignment until death), and safety.

### Statistical analysis

The MONARCH 3 study was designed to compare the investigator-assessed PFS of patients treated with abemaciclib plus a nonsteroidal AI versus placebo plus a nonsteroidal AI. The primary statistical analysis of PFS was performed on the intent-to-treat (ITT) population defined as all patients randomized to study treatment. A sensitivity analysis was planned to assess the PFS based on a fully blinded, independent central review. Power calculations and methods for analyzing the primary and secondary endpoints were previously reported.^21^ Subgroup analyses were assessed on subgroups pre-specified in the protocol.

Exploratory analyses of duration of response and change in tumor size were performed. PBRF which incorporates duration of response and ORR, was calculated using the method of Temkin.^27^ Using the method of Ellis et al.^22^, EDoR was calculated as the product of the fraction of patients with a response and the mean duration of response in responding patients for each arm assuming a Weibull distribution. The treatments were formally compared based on the ratio of EDoR, which follows a standard normal distribution on the log scale. Change in tumor size over time was compared using a mixed model with an unstructured covariance matrix.

To evaluate the association between early toxicity and efficacy, a landmark analysis was performed for diarrhea. A landmark was selected based on the median time to onset for diarrhea. Patients were stratified by whether diarrhea had or had not been observed by the landmark, and PFS was compared between each of these groups and the placebo arm using a Cox model.

To examine the impact of dose reductions on efficacy, a time-dependent covariate analysis of dose level versus PFS was performed. The dose covariate for all patients was initially set at the study starting dose of 150 mg for all patients and was adjusted at a patient level as physician-directed dose modifications were reported. For patients who discontinued abemaciclib completely prior to progression, the covariate was set to 0 mg from discontinuation to progression.

## Supplementary information

Supplementary Information

## Data Availability

Lilly makes patient-level data available from Lilly-sponsored studies on marketed drugs for approved uses following acceptance for publication. Lilly is one of several companies that provide this access through the website clinicalstudydatarequest.com. Qualified researchers can submit research proposals and request anonymized data to test new hypotheses. Lilly’s data sharing policies are provided on the clinicalstudydatarequest.com site under the Study Sponsors page.

## **Supplementary information**

**Supplementary information** accompanies the paper on the *npj Breast Cancer* website (10.1038/s41523-018-0097-z).
