# Supplementary material for: MONARCH 3 final PFS: a randomized study of abemaciclib as initial therapy for advanced breast cancer
Source: NPJ Breast Cancer. 2019 Jan 17;5:5. doi: 10.1038/s41523-018-0097-z (PMC6336880; doi:10.1038/s41523-018-0097-z)
Supplement: Supplementary file 1 — Supplementary Information [file 41523_2018_97_MOESM1_ESM.pdf]

Supplementary Table S1. Discontinuation due to AEs (safety population)

>1 patient in any arm	abemaciclib plus nonsteroidal AI (n = 327) n (%)	placebo plus nonsteroidal AI (n = 161) n (%)
**Patients Discontinued Any Study Drug due to AE**	**82 (25.1)**	**7 (4.3)**
Neutropenia	9 (2.8)	0
ALT increased	7 (2.1)	0
Lung infection	7 (2.1)	0
Diarrhea	6 (1.8)	0
Nausea	5 (1.5)	0
Chronic kidney disease	4 (1.2)	0
Embolism	4 (1.2)	0
Anemia	3 (0.9)	0
Blood creatinine increased	3 (0.9)	0
Dyspnea	3 (0.9)	0
Lymphopenia	2 (0.6)	0
Thrombocytopenia	2 (0.6)	0
AST increased	2 (0.6)	0
Pneumonitis	2 (0.6)	0
Pulmonary fibrosis	2 (0.6)	0
Weight decreased	2 (0.6)	0
Rash	2 (0.6)	0
**Patients Discontinued All Study Treatment due to AE**	**54 (16.5)**	**5 (3.1)**
Alanine aminotransferase increased	6 (1.8)	0
Lung infection	6 (1.8)	0
Diarrhea	4 (1.2)	0
Embolism	4 (1.2)	0
Neutropenia	3 (0.9)	0
Aspartate aminotransferase increased	2 (0.6)	0
Weight decreased	2 (0.6)	0
Thrombocytopenia	2 (0.6)	0
Dyspnea	2 (0.6)	0
Pulmonary fibrosis	2 (0.6)	0
Chronic kidney disease	2 (0.6)	0

Note: Discontinuation of study treatment means that abemaciclib or placebo and NSAI were discontinued.

Supplementary Table S2. Dose reductions for patients who discontinued abemaciclib or placebo due to non-fatal AEs

**Patients who discontinued due to non-fatal AEs[a], *n* (%)**	**abemaciclib plus nonsteroidal AI (*n* = 74)**	**placebo plus nonsteroidal AI (*n* = 3)**
Number of dose reductions		
0	37 (50.0)	2 (66.7)
1	14 (18.9)	1 (33.3)
2	23 (31.1)	0 (0.0)

[a]Fatal AEs were not included in the analysis.
Abbreviation: AEs, adverse events.

Supplementary Table S3. Dose reduction level versus PFS

Reduced dose vs protocol dose (150 mg[a])	Events (Alternative)	Events (Reference)	HR	95% CI	*P*[b]
100 mg vs 150 mg	22	62	0.764	0.467-1.251	0.2849
50 mg vs 150 mg	11	62	0.985	0.511-1.902	0.9650

[a]Reference dose
[b]Wald's test

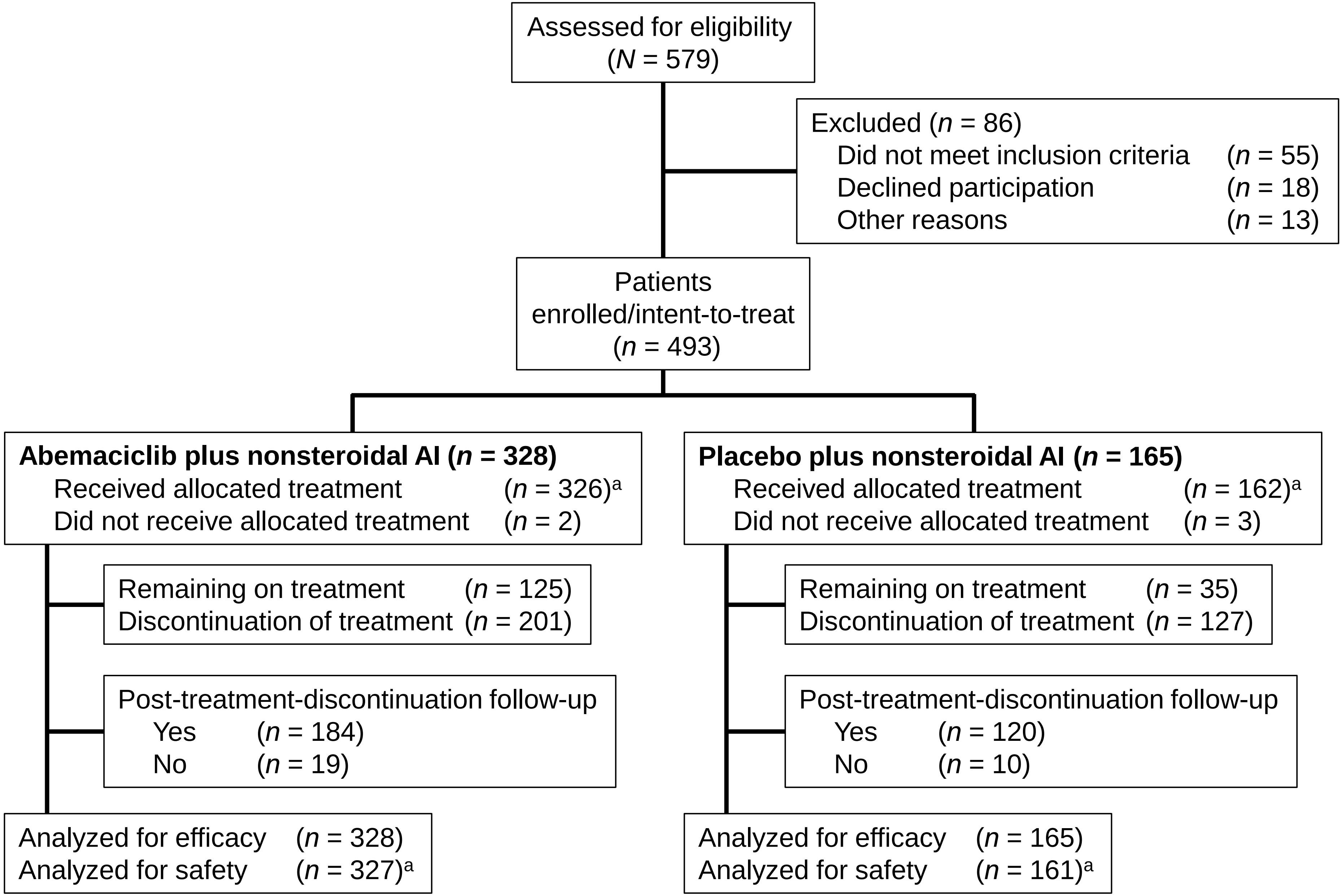

Supplementary Figure S1. CONSORT flow diagram.[21] [a]One patient who was randomized to the placebo arm received abemaciclib treatment in the first cycle and was counted in the safety population of the abemaciclib arm.

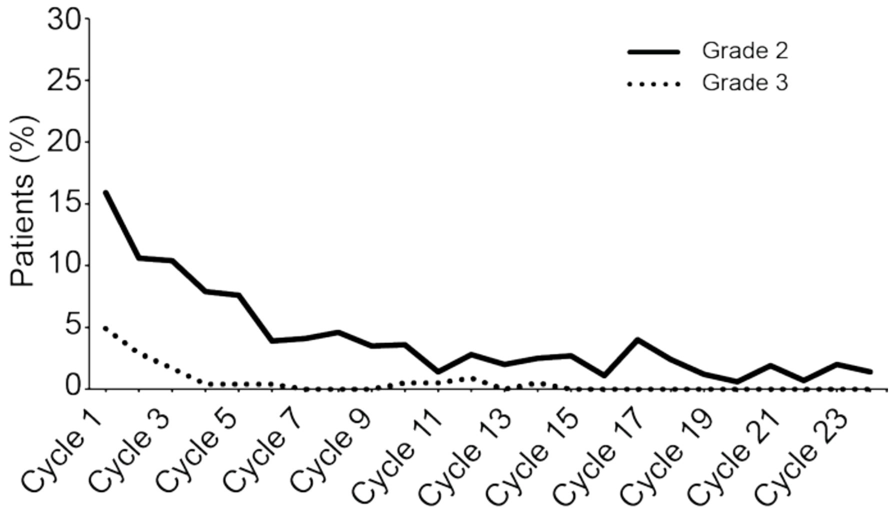

Supplementary Fig. S2.
Patients with Grade 2 or 3 diarrhea by cycle in the abemaciclib arm.

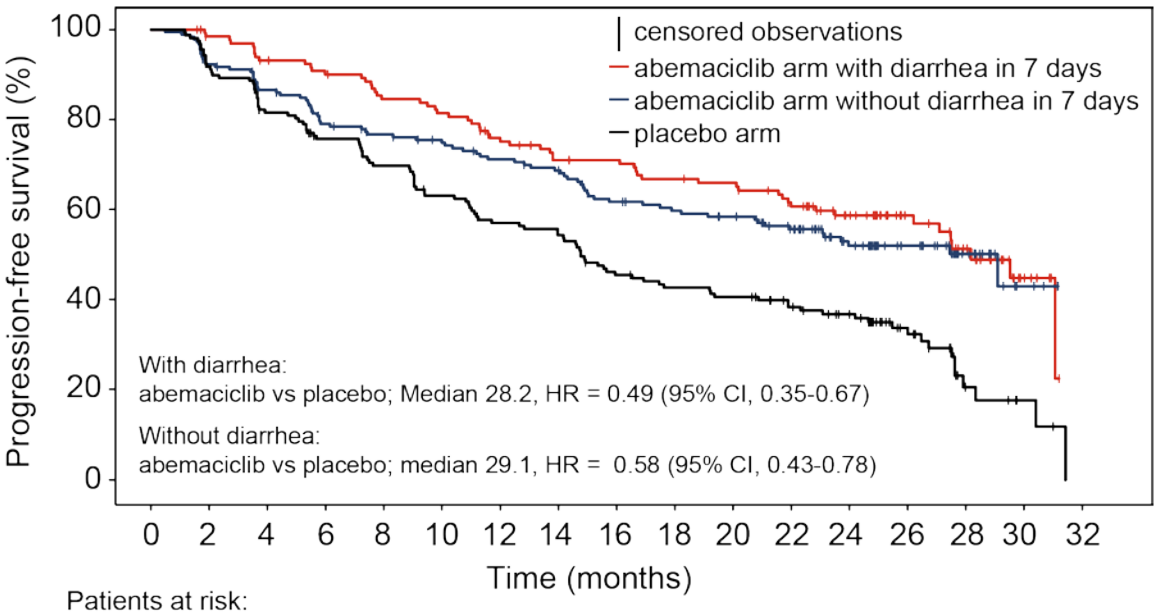

Patients at risk:

abemaciclib + nonsteroidal AI with diarrhea

134 131 122 117 109 105 94 88 86 80 78 69 53 34 23 8

abemaciclib + nonsteroidal AI without diarrhea

183 165 152 136 129 123 116 111 97 92 88 78 55 36 19 4

placebo + nonsteroidal AI

159 145 127 115 106 96 85 82 67 62 59 52 43 25 8 4

Supplementary Fig. S3.

Relationship between early diarrhea and PFS. PFS of patients with or without diarrhea (any grade) within the first 7 days of treatment. Note: PFS events occurring prior to day 7 were excluded from the analysis.
